# Upright versus supine MRI: effects of body position on craniocervical CSF flow

**DOI:** 10.1186/s12987-021-00296-7

**Published:** 2021-12-24

**Authors:** Marco Muccio, David Chu, Lawrence Minkoff, Neeraj Kulkarni, Brianna Damadian, Raymond V. Damadian, Yulin Ge

**Affiliations:** 1 Bernard and Irene Schwartz Center for Biomedical Imaging, Department of Radiology, NYU Grossman School of Medicine, 660 First Avenue, 4th floor, New York, NY 10016 USA; 2 FONAR Corporation, Melville, NY USA

**Keywords:** Cerebrospinal fluid, Neurofluids, Body position, CSF hydrodynamics, Waste clearance, Central nervous system

## Abstract

**Background:**

Cerebrospinal fluid (CSF) circulation between the brain and spinal canal, as part of the glymphatic system, provides homeostatic support to brain functions and waste clearance. Recently, it has been observed that CSF flow is strongly driven by cardiovascular brain pulsation, and affected by body orientation. The advancement of MRI has allowed for non-invasive examination of the CSF hydrodynamic properties. However, very few studies have addressed their relationship with body position (e.g., upright versus supine). It is important to understand how CSF hydrodynamics are altered by body position change in a single cardiac phase and how cumulative long hours staying in either upright or supine position can affect craniocervical CSF flow.

**Methods:**

In this study, we investigate the changes in CSF flow at the craniocervical region with flow-sensitive MRI when subjects are moved from upright to supine position. 30 healthy volunteers were imaged in upright and supine positions using an upright MRI. The cranio-caudal and caudo-cranial CSF flow, velocity and stroke volume were measured at the C2 spinal level over one cardiac cycle using phase contrast MRI. Statistical analysis was performed to identify differences in CSF flow properties between the two positions.

**Results:**

CSF stroke volume per cardiac cycle, representing CSF volume oscillating in and out of the cranium, was ~ 57.6% greater in supine (p < 0.0001), due to a ~ 83.8% increase in caudo-cranial CSF peak velocity during diastole (p < 0.0001) and extended systolic phase duration when moving from upright (0.25 ± 0.05 s) to supine (0.34 ± 0.08 s; p < 0.0001). Extrapolation to a 24 h timeframe showed significantly larger total CSF volume exchanged at C2 with 10 h spent supine versus only 5 h (p < 0.0001).

**Conclusions:**

In summary, body position has significant effects on CSF flow in and out of the cranium, with more CSF oscillating in supine compared to upright position. Such difference was driven by an increased caudo-cranial diastolic CSF velocity and an increased systolic phase duration when moving from upright to supine position. Extrapolation to a 24 h timeframe suggests that more time spent in supine position increases total amount of CSF exchange, which may play a beneficial role in waste clearance in the brain.

## Background

Cerebrospinal fluid (CSF) plays an important role in providing structural and homeostatic support [1] to the central nervous system (CNS) especially through a constant supply of nourishment and toxic waste clearance [2]. Recent studies have indicated the importance of CSF within the glymphatic system. The modern model, still source of debate, hypothesizes that CSF flows within the subarachnoid space surrounding the brain, exchanging nutrients as well as clearing waste out of the extracellular interstitial space and into the venous blood via the arachnoid villi, or via the glymphatic system [3, 4]. This highlights the strong connection between CSF circulation and brain waste clearance.

To further support such close connection, CSF movement has been shown to be strongly driven by arterial pulsation [5, 6], whose waveforms propagate throughout the brain and surrounding CSF. Increased arterial pulsation frequency has been linked to observed larger amounts of CSF flowing into the intracranial space, and the opposite was observed when such pulsation was damped [7]. Furthermore, recent studies have shown that CSF flow is bidirectional in the subarachnoid space at the cervical spinal levels, following the waveforms generated by the heartbeat. More specifically, CSF has been reported to flow from the spinal canal to the intracranial space during diastole and out of the cranium during systole [8, 9].

The advancement of MRI has furthered traditional studies of CSF flow evaluation, allowing non-invasive examination of pulsatile hydrodynamic properties. However, very few studies have addressed the changes in CSF hydrodynamics that might follow a shift in body position. It is well established that moving from an upright to a supine position can cause the decrease of the heart rate (HR) [10–12]. Such body position change can even cause a physical shift in brain structures [13]. Based on previous similar studies, we therefore expect that such body position changes will affect the CSF flow. A study by Alperin et al. [14] provides clear and comprehensive insights on how body orientation affects cerebrovascular properties. This study showed a greater arterial cerebral blood flow in subjects placed in the supine position compared to upright, together with greater venous blood outflow. The literature on CSF flow changes due to body position shift, however, is sparse and limited by either small, unisex samples or animal or synthetic models.

In this light, it is important to understand how postural changes alter CSF hydrodynamics; especially since the underlying biophysiological mechanisms are still poorly understood. Studies have reported that CSF flow along the perivascular space and via the glymphatic pathway may play a crucial role in maintaining brain functions and waste clearance in aging and age-related dementia, as extensively reviewed by Simon and Iliff [15] and recently by Rasmussen et al. [16]. It is also possible to address the effects that time spent in the supine position (mimicking sleep) can have on CSF circulation even in healthy human subjects, as few recent studies have reported [17, 18].

Hence, the purpose of this study was to investigate changes in CSF flow and volume exchange at the C2 cervical spinal subarachnoid space when the body position of healthy subjects is changed from upright to supine using flow-sensitive MRI.

## Methods

MRI scans from 30 asymptomatic volunteers (mean age = 47 ± 15 years; 9 males, 21 females) were studied retrospectively. Scans were obtained from the research and development database of the MRI device manufacturer (FONAR, Melville, New York) between April 8, 2014 and May 2, 2016. Volunteers in this database were scanned for the purpose of internal research and CSF flow reference. Originally, 65 subjects were enrolled and scanned. Scans were subsequently excluded from the study based on the following exclusion criteria: (1) evidence of neurologic symptoms and evident spinal abnormalities (e.g. cervical spondylosis, loss of cervical lordosis or scoliosis (N = 16), identified on T2 and T1-weighted MRI images, (2) Poor image quality, as screened by three independent readers, for imaging slice placement as well as artifacts such as velocity aliasing and movement (N = 19). If either supine or upright images did not pass the screening, the corresponding subject’s data was entirely excluded from further analysis. Written informed consent was obtained at the time of the appointment. This retrospective study, and the scan data, was both initially recorded and reviewed in a de-identified manner with no personal health identifiers attached to it. Therefore this study is IRB exempt.

**Fig. 1:**
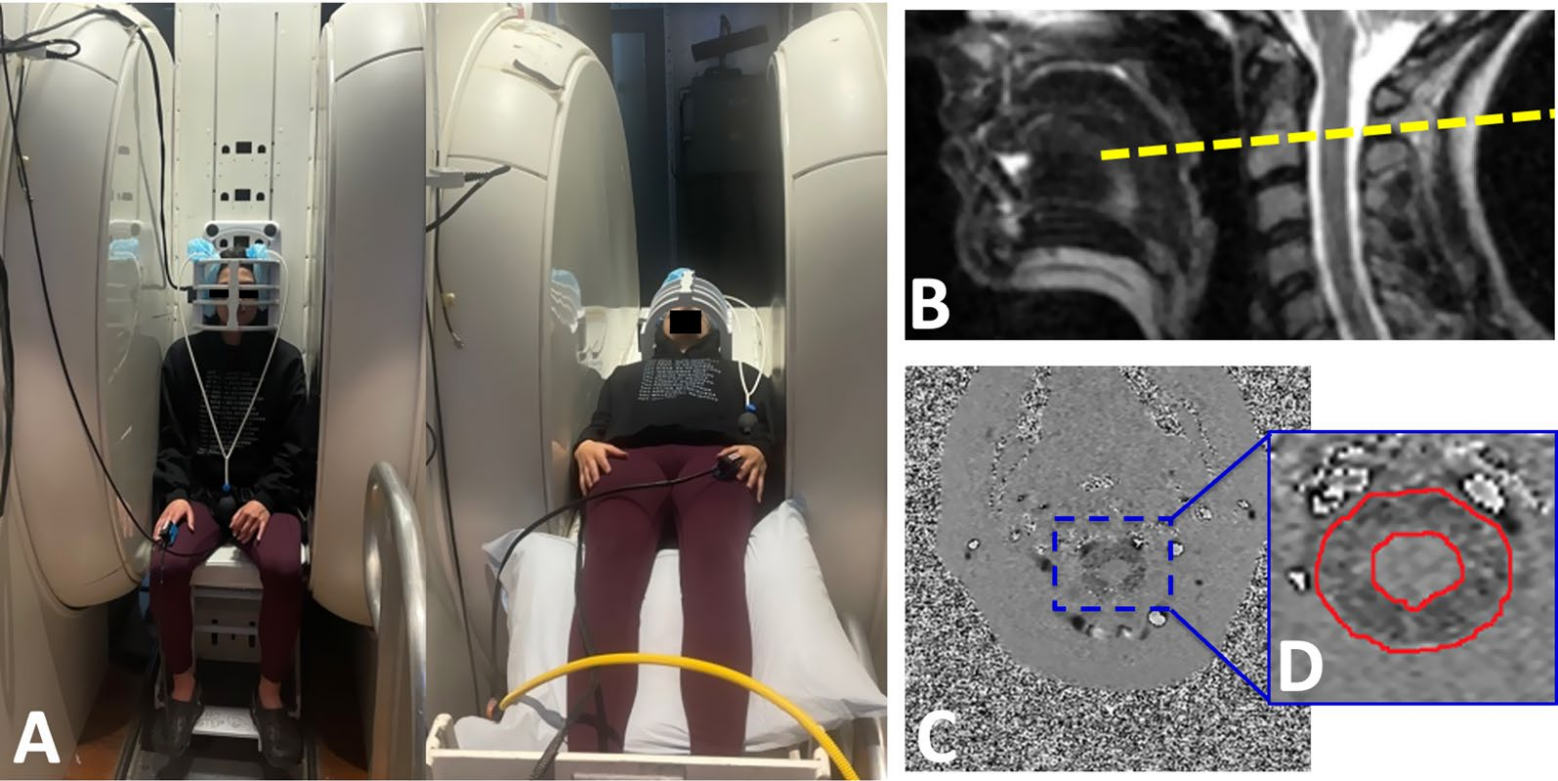
Experimental set up. **A** Body positions during MR scan, example of a subject being scanned in upright seated position and then moved to a supine position within an upright MR scanner. **B** Location of phase-contrast imaging axial slice at the mid-C2 level (yellow dotted line) used to image CSF flow and spinal cord pulsation. **C** Phase image from the phase-contrast imaging depicted in the previous image. **D** Region of interest (ROI) drawn manually to delineate the CSF in the spinal canal excluding the spinal cord on the phase image

All the MRI scans were performed on a 0.6T MRI, which allows images to be obtained with the subject in both upright and supine body positions. A quadrature craniocervical junction coil was used to image the foramen magnum and upper cervical spine region. As shown in Fig. 1A, MRI protocols were performed firstly in the seated upright followed by horizontal supine position. A 10 min interval separated the upright and supine acquisition, in order to reposition the MRI table. Sagittal T2 and axial T1 anatomical scans were acquired in the upright position. CSF flow and spinal cord pulsation were imaged using axial cine phase-contrast MRI (PC-MRI) at the mid-C2 level and perpendicular to the spinal canal (velocity encoding along the slice-select direction) (Fig. 1B). The pulse sequence was based on a RF-spoiled gradient echo sequence with TR = 21 ms, TE = 11.5 ms, slice thickness = 8 mm, flip angle = 20°, matrix = 256 × 128 up-scaled to 256 × 256, NEX = 2, and FOV = 16 cm. To visualize the overall CSF flow pattern, a large FOV (26 cm) single slice was imaged in the midline sagittal plane (velocity encoding along the readout superior-inferior direction). Velocity encoding (VENC) parameter was set to 5 cm/s, which represents the average CSF velocity at the cervical spinal level and has been previously used in other studies.

Throughout the scanning session, cardiac blood flow waveform was measured with a photo plethysmograph (FONAR, Melville, New York) placed on the subject’s finger. This was then used to register each cardiac cycle and to reconstruct the MRI data into 32 images over the cardiac cycle; a technique commonly referred to as retrospective MRI gating.

After the images were acquired, quantification of CSF flow and spinal cord pulsation was accomplished by manually drawing regions of interest (ROI) in the spinal canal around the spinal cord on the axial mid-C2 PC-MRI scan (Fig. 1C, D). The average pixel intensity over the CSF canal and spinal cord was used to calculate the average velocity of CSF and spinal cord motion, with any phase offset corrected by subtracting the spinal cord net phase change over the whole cardiac cycle.

**Fig. 2:**
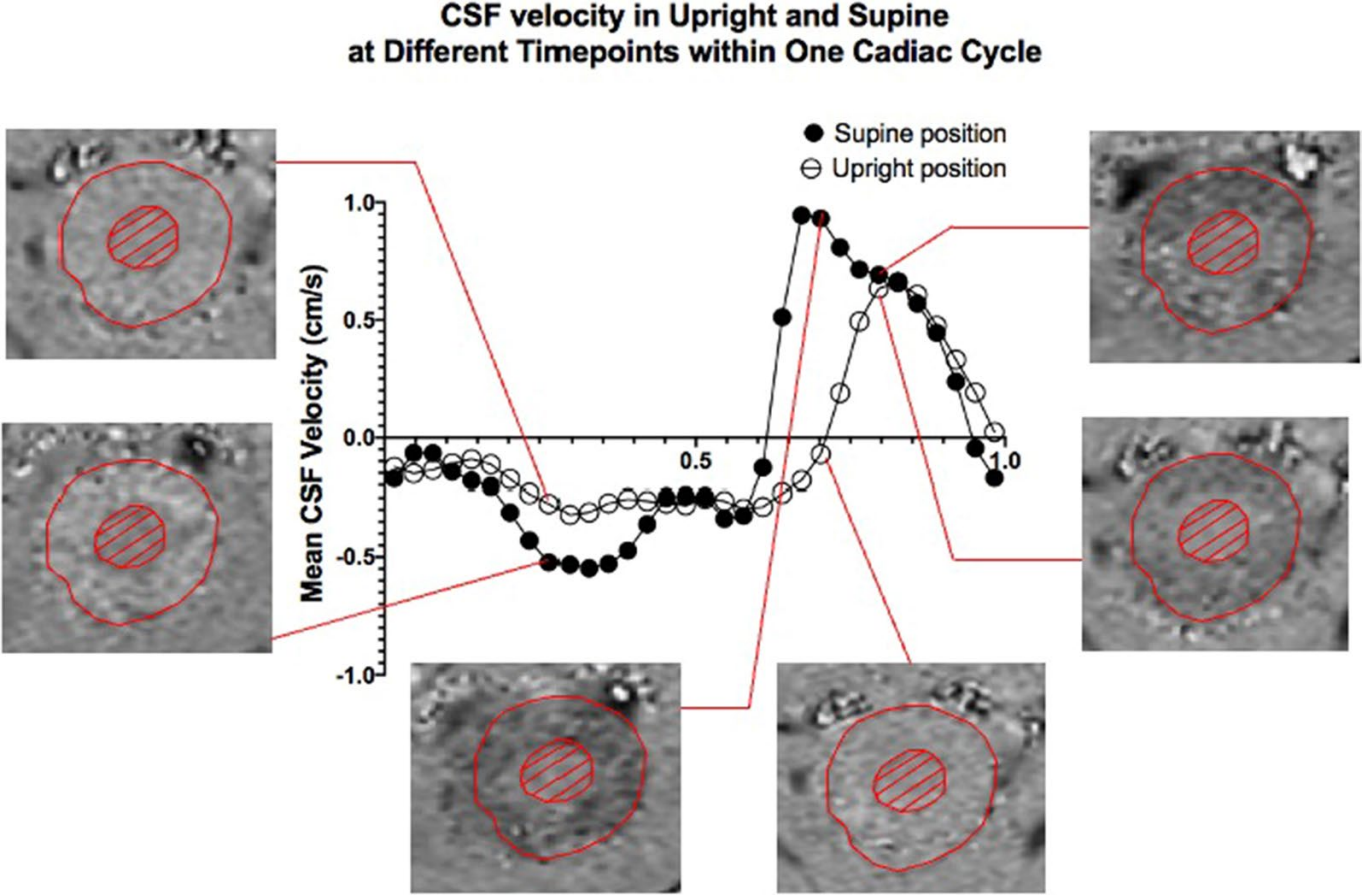
Mean CSF velocity measurements over one cardiac cycle. Representative CSF velocity changes in one subject over one cardiac cycle as measured at mid-C2 in a volunteer during upright and supine body orientations. Notice the wider systolic peak during systole (positive values) in supine compared to the narrower one observed in upright posture. Furthermore, the magnitudes of CSF velocities appear to be consistently lower in upright compared to supine (bottom images), indicated by the CSF signal intensities on the phase image (red circle of spinal canal excluding cord). Note that the dark intensity is cranio-caudal whilst bright intensity is caudo-cranial direction

The volume flow through the CSF canal was derived by multiplying the CSF velocity with the area of pericord CSF canal. Figure 2 shows a typical plot of CSF velocity through C2 craniocervical level over one cycle in the upright and supine positions, with examples of PC-MRI images for specific time points.

From the velocity and flow rate time curves, various parameters can be extracted (Tables 1 and 2). CSF flow was extracted from the area under the mean flow curve for diastole and systole separately. In contrast to the flow directions in cardiac phases, the systolic phase here refers to the CSF flow in the positive cranio-caudal direction while diastolic phase refers to the negative caudo-cranial direction. Furthermore, the stroke volume, which was defined as bi-directional CSF volume exchanged through C2 level over one cardiac cycle, was calculated by taking the smaller of the area under the CSF flow curve during CSF systole and diastole (Fig. 2). From this last measurement, an hourly rate of CSF volume exchanged was extrapolated by multiplying the stroke volume with the subject-specific heart rate measured. Two scenarios were then established, representing hypothetical cumulative stroke volumes over a 24-h period. The first scenario consisted of 10 h spent in the supine position (mimicking sleeping posture in young population) and 14 h in upright position (mimicking awake posture); the second was composed of 5 h in supine (mimicking sleeping posture in the elderly) and 19 h in the upright position. The goal of this extrapolation analysis is to allow for real-life translation of the results (e.g. aging effects or sleep deprivation) and support the need for further investigation.

**Table 1:** CSF temporal properties measured in supine and upright position

Measurements	Group Average		%Change from Upright	Paired t-test (p-value)
	Upright	Supine		
Heart rate (BPM)	76.09 ± 9.50	68.89 ± 10.83	− 9.5	8.43E−05
Systolic fractional duration (%Cycle)	30.9 ± 4.5	37.7 ± 6.4	22.6	1.89E−06
Diastolic fractional duration (%Cycle)	69.1 ± 4.5	62.3 ± 6.4	− 10.1	2.05E−06
Systolic duration (s)	0.25 ± 0.05	0.34 ± 0.08	36.0	1.06E−07
Diastolic duration (s)	0.55 ± 0.08	0.56 ± 0.12	1.8	0.78

Percentage change was calculated as follows: ((supine-upright)/upright)*100

**Table 2:** CSF hydrodynamic properties measured in supine and upright

Measurements	Group Average		%Change from Upright	Paired t-test (p-value)
	Upright	Supine		
CSF Stroke volume (cm^3^/cycle)	0.33 ± 0.13	0.52 ± 0.19	57.6	6.73E−06
Total systolic CSF volume displaced (cm^3^/cycle)	0.38 ± 0.13	0.56 ± 0.21	47.4	6.26E−06
Total diastolic CSF volume displaced (cm^3^/cycle)	− 0.35 ± 0.12	− 0.57 ± 0.19	62.9	2.46E−06
Peak systolic CSF flow (cm^3^/s)	2.79 ± 0.73	2.98 ± 0.91	6.8	0.10
Peak diastolic CSF flow (cm^3^/s)	− 0.98 ± 0.25	− 1.71 ± 0.48	74.5	1.70E−08
Peak systolic CSF velocity (cm/s)	1.06 ± 0.32	1.16 ± 0.35	9.4	0.16
Peak diastolic CSF velocity (cm/s)	− 0.37 ± 0.13	− 0.68 ± 0.24	83.8	1.06E−07
Area of CSF space (cm^2^)	2.71 ± 0.58	2.64 ± 0.68	− 2.6	0.48

Percentage change was calculated as follows: ((supine-upright)/upright)*100

A two-tailed paired Student’s t-test was used to determine statistically significant differences in CSF hydrodynamic parameters between the upright and supine positions. Percentage change between upright and supine measurements for each subject was calculated using the following equation: ((supine-upright)/upright)*100.

## Results

**Fig. 3:**
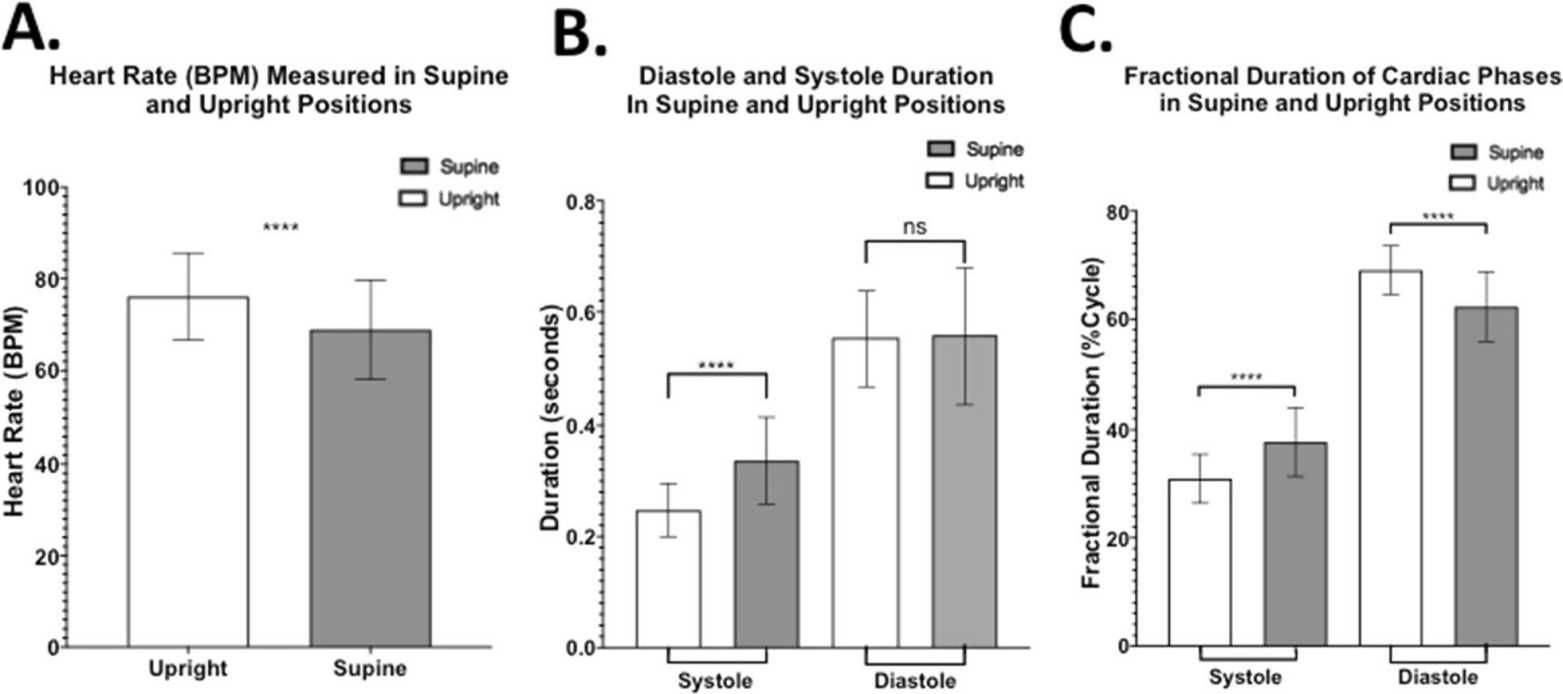
CSF temporal properties changes when moving from upright to supine. **A** Heart rate (HR) values plotted for the whole sample show significantly higher HR in upright compared to supine position. **B** Duration of each CSF phase (diastole or systole) measured within one cardiac cycle in supine and upright position showing in general the CSF diastolic phase is longer than the CSF systolic phase. Notice the significant increase in systolic duration in supine but unchanged diastolic duration. **C** Fractional duration of systole or diastole in a single cardiac cycle, showing systole covering a larger part of the cycle in supine, at the expenses of the diastolic coverage. [**** = p < 0.0001; ns = not statistically significant]

Change in position from upright to supine resulted in a significant decrease in heart rate (HR) (p < 0.0001). HR was ~ 9.5% slower in the supine position (Fig. 3A). To further investigate the specific changes within one cardiac cycle, initiated by the observed changes in HR between the two body positions, systolic and diastolic durations were analyzed. Significant changes in duration were observed only during systole (Fig. 3B). In this phase of the cycle, a significant increase in duration was observed from upright (0.25 ± 0.05 s) to supine (0.34 ± 0.08 s; p < 0.0001) position; in other words, the systolic time window for cranio-caudal CSF flow is significantly increased from upright to supine body position while there is no significant change in CSF diastolic duration between the two postures.

The individual contributions of systole and diastole to the cardiac cycle and their relationship to the heartbeat were further analyzed. To allow comparison between the different body positions, the data was normalized by looking at how much of a single cycle each phase covers (fractional duration). In the upright position, systole made up about 30.9 ± 4.5% of the cycle on average, and diastole the remaining 69.1 ± 4.5%. In the supine position, there was a ~ 22.6% increase in systolic fractional duration compared to upright, bringing it to 37.7 ± 6.4% of the cycle in supine, which can potentially lead to the increased stroke volume. This was compensated by a ~ 10.1% decrease, from what measured in upright, of diastolic fractional duration, which then made up circa 62.3 ± 6.4% of the cycle in supine. Such changes were all statistically significant (p < 0.0001) and are represented in Fig. 3C.

**Fig. 4:**
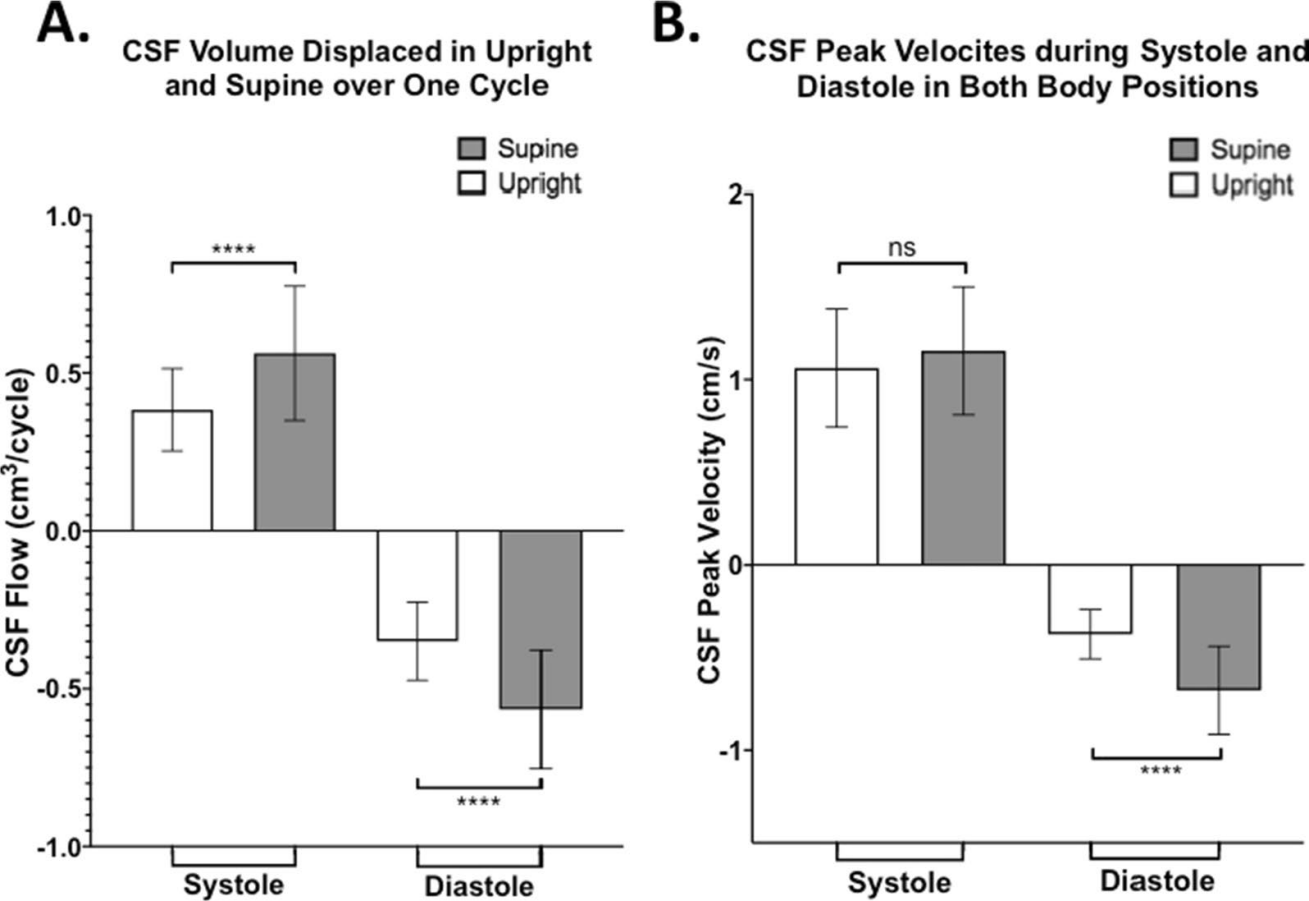
Changes in CSF hydrodynamic properties due to body position shift. **A** CSF volume exchanged at craniocervical level during one cardiac cycle (systole or diastole) in both body positions. Notice the difference in directionality. **B** CSF peak velocity measured during diastole and systole in supine and upright positions. Notice how significant difference in CSF peak velocity is observed between supine and upright position in diastole (**** = p < 0.0001) but are not significantly different in systole (ns = not significant)

To better understand CSF hydrodynamics, CSF properties were divided into systolic and diastolic components and then individually compared between the two body positions. Over one cardiac cycle, we observed that CSF flow and velocity have negative values (caudo-cranial direction) during diastole and positive values (cranio-caudal direction) in systole, as plotted in Fig. 4. This subdivision also led to the observation of a significant increase of total CSF flow during both systole and diastole when moving from upright to supine. In fact, in the supine position, both systolic (0.56 ± 0.21cm^3^/cycle) and diastolic (− 0.57 ± 0.19cm^3^/cycle) CSF volume displaced at C2 spinal level was higher in magnitude compared to what was measured in the upright position (0.38 ± 0.13cm^3^/cycle and − 0.35 ± 0.12cm^3^/cycle, respectively; p < 0.0001) (Fig. 4A). To investigate the cumulative effects, we also calculated CSF volume displaced per minute, by multiplying for subject-specific HR. A significant increase in supine compared to upright was still observed in both diastole (upright = − 26.02 ± 7.84 cm^3^/min, supine = − 38.67 ± 12.18 cm^3^/min, p = 0.00004) and systole (upright = 28.45 ± 7.96 cm^3^/min, supine = 37.88 ± 12.50 cm^3^/min, p = 0.00009). CSF peak velocity during diastole, in the caudo-cranial direction, significantly increased from − 0.37 ± 0.13 cm/s in upright to − 0.68 ± 0.24 cm/s in the supine position (p < 0.0001). However, no significant changes in CSF peak systolic velocity were observed between supine (1.16 ± 0.35 cm/s) and upright (1.06 ± 0.32 cm/s; p = 0.16) positions (Fig. 4B). The area of the CSF canal in the spinal cord was also analyzed and no significant changes between the two positions were observed.

**Fig. 5:**
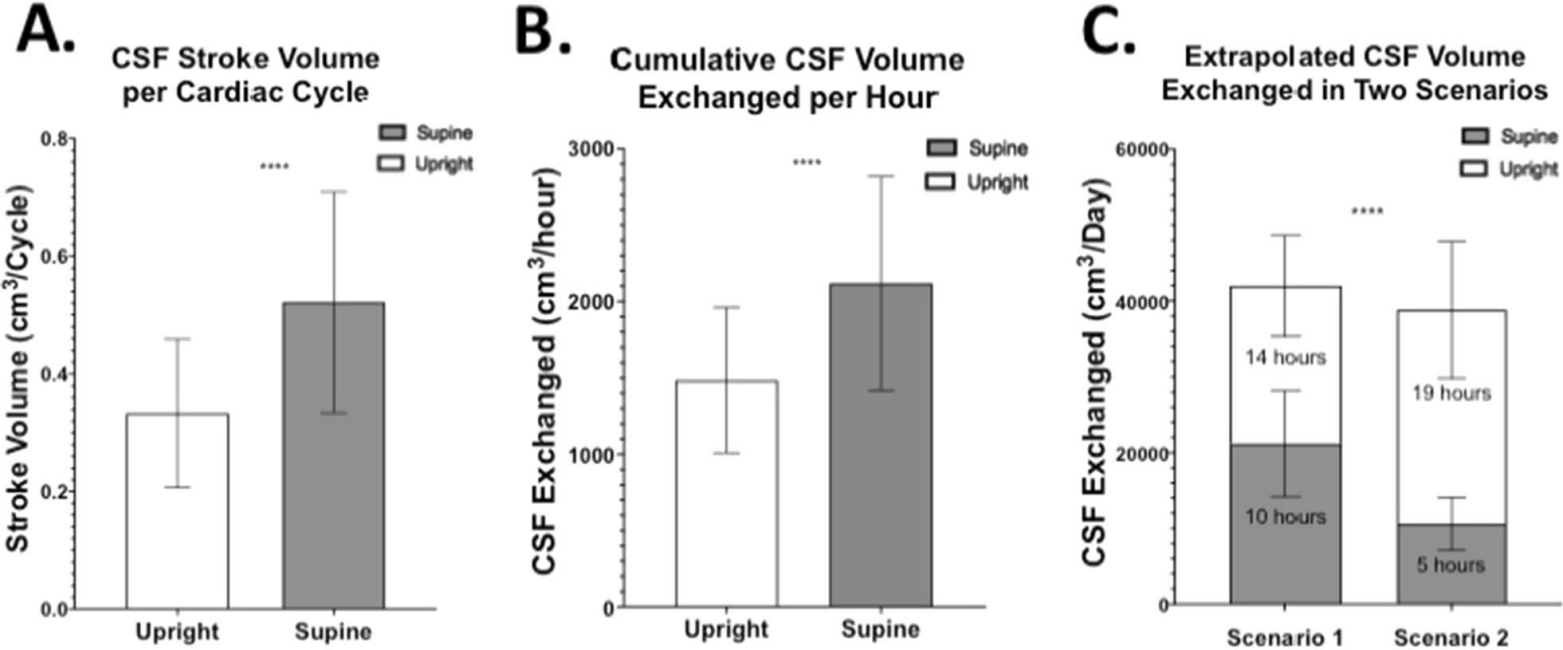
Extrapolation of stroke volume results into different timeframes. **A** Bar plot representing the CSF stroke volume exchanged between the intracranial and spinal space over one cardiac cycle in supine and upright position. **B** Extrapolation of resulting CSF volume exchanged per hour in both body orientations. **C** Extrapolation of cumulative CSF exchanged per day of supine and upright orientations in two scenarios with the first one consisting of 10 h in the supine position and 14 h in the upright position that showed significant greater (~ 7.5%) CSF volume exchanged than what obtained in the second scenario (5 h in supine and 19 h in upright position; **** = p < 0.0001)

Stroke volume, defined as CSF volume exchanged between the spinal and intracranial space over a single cardiac cycle, was ~ 57.6% greater in supine compared to upright (Fig. 5A). To investigate the cumulative effects of stroke volume, the single cardiac cycle measurements were first translated in a per minute timescale by using the subject-specific HR. The results showed same significant increase in the CSF volume exchange in supine (35.31 ± 11.67 cm^3^/min) compared to upright (24.75 ± 7.93 cm^3^/min). A per hour rate was then extrapolated and showed similar significant patterns of change, with a ~ 42.7% increase of CSF exchanged in supine (2118.67 ± 700.35 cm^3^/hour) compared to what measured in upright position (1484.97 ± 475.58 cm^3^/hour) (Fig. 5B). Figure 5C shows the results of two scenarios created to facilitate real-life implications of our findings. The first scenario shows the resulting cumulative exchange of CSF volume over one day, during which 10 h were spent in the supine position and 14 h in upright position (e.g., normal sleeping pattern of younger population). The second one shows the same extrapolated measurements but, this time, assuming 5 h in supine and 19 h spent upright (e.g. sleeping pattern of elderly population). Significantly less CSF volume is exchanged in the second scenario compared to the first one (p < 0.0001). In this example, in the 10 h spent supine scenario 7.5% more CSF is exchanged over a single day compared to the scenario in which only 5 h of the day are spent in the supine position. The CSF volumes exchanged in the first scenario were 21,186.74cm^3^ and 20,789.61cm^3^ for the 10 h spent in supine and 14 h in upright respectively, for a total of 41,976.35cm^3^ CSF exchanged in the first scenario. In the second scenario only 38,807.84 cm^3^ of CSF was exchanged over the whole day, with 10,593.37cm^3^ during the 5 h spent in supine and 28,214.47cm^3^ during the 19 h spent upright. By comparing the contribution of each body position to the total CSF stroke volume, normalized by dividing for the total CSF volume exchanged in the first scenario, we observed that a five hours reduction in time spent supine (first to second scenario) contributes to a 25.2% decrease of CSF exchanged, whilst the extra 5 h spent upright only increase the CSF exchange by 17.7% of the total stroke volume.

**Fig. 6:**
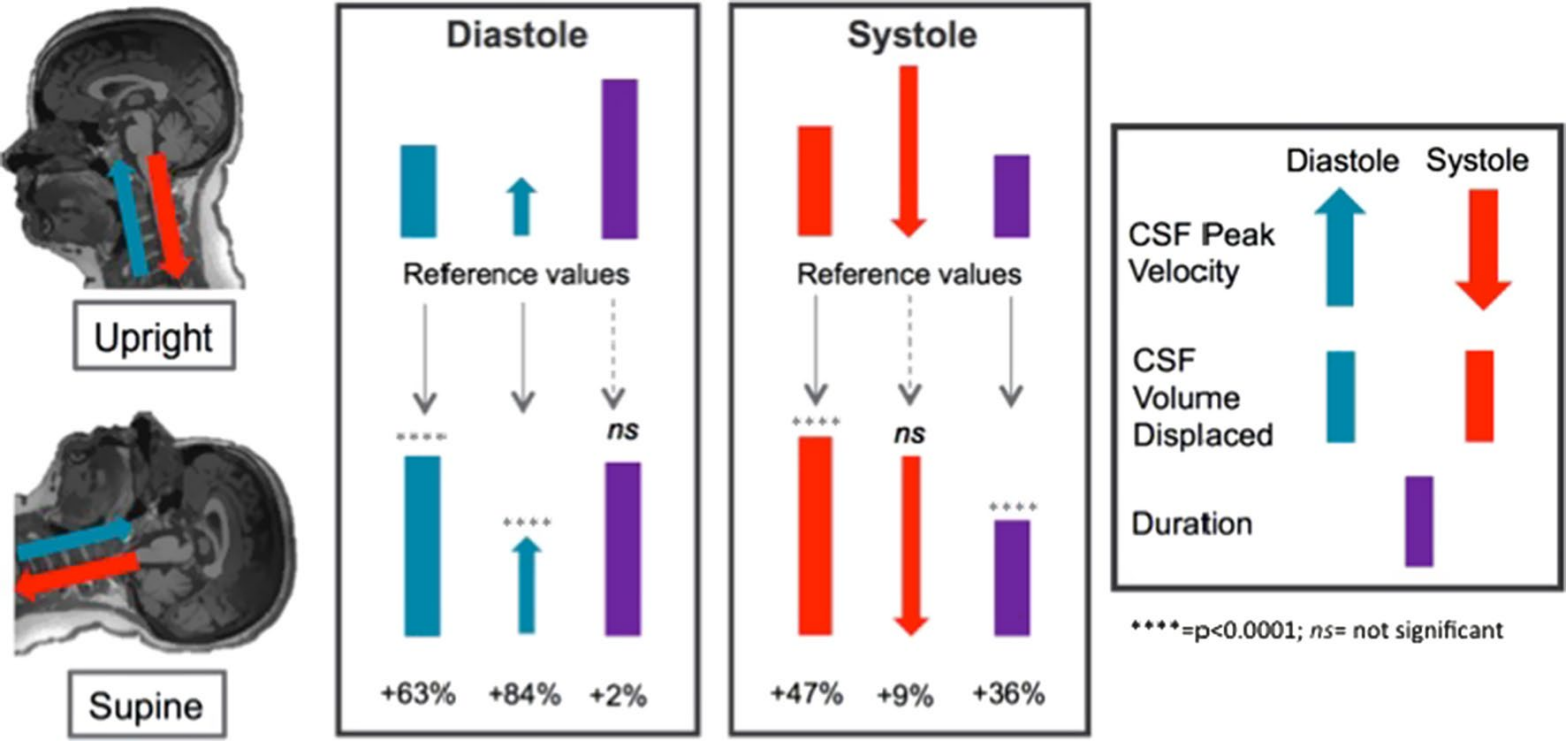
Summary of CSF hydrodynamic changes following shift from upright to supine body position. CSF peak velocities (arrow) and CSF volume displaced (bar) are distinguished in diastole and systole to highlight their caudo-cranial (blue) and cranio-caudal (red) directionality. Together with CSF phase duration (purple bar), all measurements are here represented in proportion to their absolute values. Percentage changes are reported with respect to upright measurements. Note that during diastole CSF peak velocity in supine is ~ 84% greater than what was measured in upright position whilst the diastolic duration is unchanged. However, during systole, the CSF peak velocity does not significantly change between positions whilst a ~ 36% increase is observed in supine systolic duration compared to upright. These differences lead to a greater CSF volume being displaced in supine compared to upright both cranially during diastole (+ 63%) and caudally during systole (+ 47%)

A cumulative representation of CSF hydrodynamic changes due to body position within one cardiac cycle, from upright (top row) to supine (bottom row), can be seen in Fig. 6. The differences of CSF volume exchange at C2 level between the two body positions are mainly due to the differences of the CSF peak velocity and systolic duration.

Three subjects were recruited and scanned three times in the upright position and then three times in the supine position, to evaluate the test–retest reproducibility. Exact same PC-MRI parameters and settings described in the methods section were used. Since the analysis was focused on within subject reproducibility, we found low coefficient of variation (CV < 10%) for CSF stroke volume measurement in all three subjects, which proves that the changes due to body position observed are significantly greater than the test–retest variation.

## Discussion

Circulation of CSF between ventricle, interstitial, and subarachnoid space, as well as between brain and spinal canal serves multiple important functions to support CNS including waste clearance through the glymphatic network. The adaptive physiologic methods used by the CNS to maintain a homeostatic balance of brain fluid movement in the setting of body position shifts have not yet been thoroughly studied, particularly with noninvasive quantitative imaging approaches such as MRI. However, few studies suggested important adaptive physiological mechanisms that the human body utilizes to maintain the CNS in constant, optimal equilibrium.

One of such mechanisms, thoroughly well-addressed in current literature, is the change in HR when moving from upright to supine positions or vice versa. In this study, we showed that subjects in the supine position exhibit a significantly slower HR compared to upright. This is in line with the current literature highlighting the importance of such conservation mechanism to ensure consistent blood perfusion of the body [10, 11]. Interestingly, we have observed a longer systolic duration measured in supine compared to upright position while there was unchanged diastolic duration when the position was shifted. From a neurophysiological perspective, studies have shown that, in vertical positions, the sympathetic nervous system has a stronger effect on regulating the heartbeat in order to compensate for gravity, whilst in supine positions this function is replaced mainly by vagal nerve activity that slows the heartbeat [11, 19].

Furthermore, we observed that during systole, CSF is mainly drained out of the cranium (positive value) whilst in diastole CSF mainly flows into the intracranial space (negative value). This CSF directionality is in line with findings reported in the literature [20–23]. Our key finding, however, is the clear difference in CSF volume exchanged between the spinal and cranial space due to body position changes, expressed as stroke volume. We found that ~ 57.6% more CSF was exchanged at the end of one cardiac cycle in the supine position compared to upright position, in line with previous results reported by Alperin et al. [24]. One study by Alperin et al. [14] noted that CSF flow closely followed the difference in arterial and venous blood flow throughout one cardiac cycle. The net blood flow between the two positions can be explained by the observed collapse of the interior jugular vein when moving to the upright position and the consequent shift of venous blood to secondary cervical venous structures, such as vertebral veins, in order to maintain optimal intracranial pressure [10, 25, 26]. This leads to less blood flowing out of the cranium in the upright position compared to supine, causing more blood to accumulate and limiting CSF inflow. The opposite is observed in the supine position, where high venous outflow is enabled via the internal jugular vein, consequently enabling more CSF to be exchanged between the cranium and the spinal canal [10].

We then looked at the two main contributors to the mentioned difference in CSF volume exchanged at C2 level: CSF velocity and phase durations of CSF flow. Interestingly, they change differently when moving from an upright to a supine position. In fact, we observed that in the supine position during diastole, CSF flows into the intracranial space at peak velocity that is ~ 83.8% higher than that in upright. On the other hand, during systole, CSF is drained out of the intracranial space but, instead of compensating for the larger CSF volume via increased efflux velocity, the systolic duration increases by about 36.0%. These alterations allow for larger CSF volumes to be exchanged in the supine versus upright position. Such largely increased CSF volume exchanged in the supine position may have important implication on brain waste clearance in dementia due to altered time spent in supine position in the elderly versus younger population.

Integrating our findings with what is available in the current literature, we thus obtain a general picture of the biophysiological adaptive and physical (e.g. gravity and brain structures shift) mechanisms of the brain in response to body position shift. When moving from an upright to supine position, parasympathetic (vagal) activation and sympathetic withdraw cause a decrease of heart rate as a consequence of the restored venous blood flow through the internal jugular veins, increasing the blood leaving the intracranial space and the cardiac preload during diastole [27]. Since the duration of the diastolic phase is not altered by the shift to a supine position, these changes are compensated by greater CSF volume being displaced into the cranium at greater velocity. The following systolic cardiac phase, in the supine position, is therefore characterized by an increased cardiac output [27], compared to the upright position, and increased blood influx into the intracranial space. This increase is compensated by a greater efflux of CSF into the spinal cord. Perhaps due to the lack of venous blood pulsation, which in the arteries is the main contributor of CSF flow, the velocity of CSF exiting the intracranial space is only slightly increased compared to the upright position. However, in order to move such greater volume of CSF out of the cranium, systolic duration is extended in the supine position compared to upright.

This overall system of adaptation not only provides constant structural support and protection of the brain, but also might affect the efficiency of homeostatic support. As summarized in Fig. 6, the cerebral-spinal CSF exchange is mainly contributed by the increase in CSF diastolic velocity towards the brain and systolic duration of CSF flow away from the brain, both of which increase the CSF volume exchanged at C2 level in the supine position. This offers insights in the benefits that spending time in such position (mimicking sleep) might have on the brain’s ability of waste clearance.

In fact, our extrapolation showed that even only one hour spent in supine position might allow for more CSF volume to be exchanged between the spinal canal and the brain compared to one hour spent upright. Translating this extrapolation to 24 h timeframe it is evident how the longer time (e.g., 10 h) spent in supine in a day might support better CSF flow in and out of the brain than shorter time (e.g., 5 h). This is due to the larger contribution of supine compared to upright to the overall CSF exchanged in one day. In our example, in fact, a reduction of 5 h in the supine position, from 10 h in the first scenario to 5 h in the second one, contributes with a 25.2% decrease of all CSF exchanged over the day. However, the same increase of hours in the upright position, from 14 in the first scenario to 19 in the second one, contributes only with a 17.7% increase of all CSF exchanged over the day. These differences between the two scenarios lead to a net increase of 7.5% CSF exchanged over a single day, an important daily change quantified for assessing the cumulating effect over a period of decades of aging. It must be noted, however, that this extrapolation does not account for many physiological factors that may vary during the course of 1 h, 24 h or even decades. In fact, recent studies have observed that exercise [28, 29], blood flow and blood pressure [6, 30, 31], as well as sleep [17, 32] and respiration [33–38] contribute to changes in CSF flow. Moreover, overlap of stroke volume measurements between consecutive cycles was not considered in this extrapolation and should be investigated in further studies.

Nonetheless, this extrapolation’s results raise interesting questions regarding the possible age-related effects of body position. The elderly population has been found to get significantly less hours of sleep over a day compared to the younger counterpart [39]. This, together with what we reported in our study, might suggest that, as older people spend less time in the supine position, less CSF is exchanged between the brain and the spinal cord, therefore affecting the efficiency with which the glymphatic system maintains brain homeostasis. Body position thus might be related to the surfacing of many age-related symptoms including dementia.

Furthermore, Studies have even suggested a link between hindered metabolic clearance through CSF or interstitial flow and pathogenesis of neurodegenerative diseases such as Alzheimer’s Disease and Multiple Sclerosis [40]. Whether CSF waste clearance efficiency is directly affected by body position as opposed to other biophysiological changes related to sleep is yet to be addressed. However, the changes in CSF hydrodynamic properties reported in our study may play a significant role in the way toxic waste is cleared out of the brain. The importance of further investigating such hypothesis is highlighted especially by recent studies reporting increase in misfolded protein accumulation in humans following one night of sleep deprivation [41], changes in glymphatic waste clearance during sleep [42], as well as a correlation between sleep and late-onset dementia [43]. These differences in CSF hydrodynamic properties between the two positions have to be seen in concert with the corresponding cardiovascular changes, as reviewed by Linninger et al. [44] and recently clinically demonstrated by Alperin et al. [45], in order to have a complete understanding of the adaptive mechanisms implemented by the human body.

One limitation of this study is that blood flow from main neck feeding arteries as well as from venous blood flow were not measured; these parameters may have provided a more comprehensive analysis. In addition, the effects of breathing on CSF flow dynamics were not measured and a recent study reported that coughing and controlled breathing can affect measurements of CSF flow [33–38]. However, such influence is not observed during regular breathing [37], and therefore should not significantly affect our results since, in study, subjects were not given any breathing instructions. Moreover, the comparison in this study is based on intra-subject or within-subject, therefore the inter-subject variations do not play a major role in our analyses. Regarding our extrapolations, the stroke volumes over a one hour and 24 h timeframes were obtained by multiplying the results over one cardiac cycle and the subject’s HR. It is evident that this simplification is merely speculative since it does not consider for HR, or other physiological parameters, variations over the given timeframes. Therefore, our conclusions merely aim to highlight the possible role that time spent in either body position might play in normal aging, expressing the pressing need for further investigation.

## Conclusion

To the best of our knowledge, the results of our MRI study on CSF hydrodynamic components in relationship with changes in body position are in line with what previously reported in the literature [10, 14, 24, 45]. The CSF hydrodynamics is quite complicated and multiple physical and physiological factors can influence its change. This study provides focused and detailed results to better understand the magnitude of the difference in both single cardiac cycle and extrapolated period of time mimicking young versus elderly subjects. Our findings, especially the decreased CSF exchange in upright posture, represent an innovative and promising indication for future studies to investigate the CSF-posture correlation in brain waste clearance and aging, as well as in a range of different neurodegenerative ailments.

## Data Availability

The data and analysis methods of this study will be made available by contacting the corresponding author via email, respecting all formal data sharing agreements.
